# Effect of stereocomplex crystal and flexible segments on the crystallization and tensile behavior of poly(l-lactide)

**DOI:** 10.1039/c8ra05355c

**Published:** 2018-08-09

**Authors:** Xiaolu Li, Xiuqin Zhang, Guoming Liu, Zhongkai Yang, Bo Yang, Yue Qi, Rui Wang, De-Yi Wang

**Affiliations:** Beijing Key Laboratory of Clothing Materials R & D and Assessment, Beijing Engineering Research Center of Textile Nanofiber, School of Materials Science & Engineering, Beijing Institute of Fashion Technology Beijing 100029 China clyzxq@bift.edu.cn clywangrui@bift.edu.cn; CAS Key Laboratory of Engineering Plastics, CAS Research/Education Center for Excellence in Molecular Sciences, Institute of Chemistry, Chinese Academy of Sciences Beijing 100190 China; IMDEA Materials Institute Madrid 28906 Spain

## Abstract

The effects of poly(ethylene glycol) (PEG) and/or poly(d-lactide) (PDLA) blocks on the crystallization and mechanical properties of poly(l-lactide) (PLLA) were investigated systematically *via* differential scanning calorimetry (DSC), polarized optical microscopy (POM), wide-angle X-ray diffraction (WAXD) and tensile testing. The structural evolution during uniaxial stretching of the obtained blends above the glass transition temperature was studied by *in situ* WAXD. It was observed that the stereocomplex (SC) crystals promoted the nucleation of PLLA homocrystals (α/α′ crystal), and flexible PEG blocks could enhance the spherulitic growth rate. The PLLA/PDLA–PEG–PDLA had a higher elongation at break than that of PLLA/PDLA without significant loss in tensile strength and stiffness. *In situ* WAXD showed that the PLLA/PDLA–PEG–PDLA crystallized faster during stretching. It was shown that the incorporation of SC crystals and flexible PEG blocks could not only accelerate the crystallization but also improve the toughness of PLLA.

## Abstract 2

Effect of stereocomplex crystal and PEG segments in blends on the crystallization and tensile behavior of PLLA.

## Graphical abstract

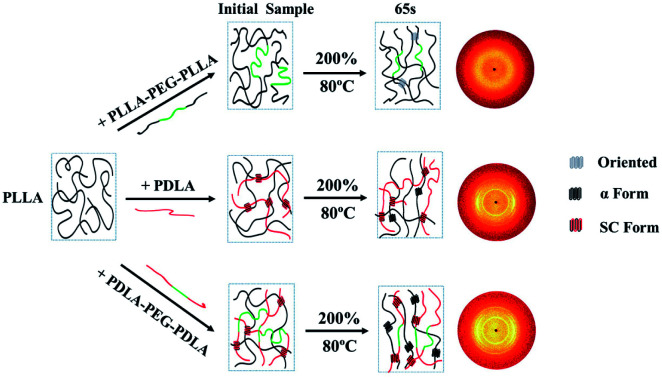

## Introduction

Poly(lactide) (PLA), one of the representative biodegradable materials, has found a vast number of applications in different areas, such as textiles, medicine and packaging.^1–3^ However, PLA possesses some intrinsic shortcomings, including low crystallization rate, poor heat resistance, and toughness, which limit its applications. It is believed that high-performance PLA materials can be achieved by adjusting the crystalline structure by processing, blending with other polymers or adding organic or inorganic additives.^4–7^

Many nanofillers have been applied to tailor the crystallization rate in PLA, such as carbon nanotubes,^8^ graphene or graphene oxide^7,9^ and nanoclay.^10^ However, those additives may raise environmental or health issues which hinder them from applications in medical areas or food packaging.

Ikada *et al.*^11^ reported that a stereocomplex (SC) crystal can be obtained by blending poly(d-lactide) (PDLA) with poly(l-lactide) (PLLA). With a melting point 50 °C higher than that of PLA homocrystals (HC), SC crystals provide a promising solution to heat resistant PLA materials.^12^ More importantly, SC crystals can effectively mediate the crystallization rate of PLA while maintaining the biocompatibility. Anderson *et al.*^13^ found that the nucleating efficiencies increased by up to about 100% by adding 3 wt% of PDLA into PLLA, which suggested the nucleation effect of SC crystals was better than that of talc. Besides, Yin *et al.*^14^ also showed that PDLA had better nucleation efficiency than talc for PLLA under static and stretching condition. Tsuji *et al.*^15^ demonstrated that the crystallization rate and nucleation density of PLLA α crystals could be significantly increased with the aid of a small amount of SC crystals.

Although SC crystals could promote the crystallization of PLLA by heterogeneous nucleation,^16^ the toughness is still quite low for PLLA with high crystallinity. Introducing flexible segments including poly(ε-caprolactone) (PCL),^17^ poly(butylenes succinate) (PBS),^18,19^ poly(butylene succinate-*co*-adipate) (PBSA)^20^ and poly(ethylene glycol) (PEG)^21–24^ into PLLA is an effective way to improve the toughness. However, blending PLLA with flexible polymers generally results in a significant decrease in modulus, tensile strength and thermal deflection temperature of PLLA. To settle these inevitable shortcomings, new strategies which could both improve crystallization rate and toughness are favoured.

It has been found that the introduction of PDLA block copolymers with soft chains as the modifier of PLLA could enhance the thermal and mechanical stability of PLLA without compromising the stiffness and tensile strength.^21,25^ Liu *et al.*^26^ reported that the tensile strength and elongation at break of PLLA were effectively improved when the content of PDLA–PEG–PDLA block copolymer reached up to 30 wt%. In our previous work, the effect of the content of PDLA–PEG–PDLA on the properties of PLLA has been investigated and the tensile elongation at break of PLLA could reach up to 200% at 50 °C.^27^ Song *et al.*^28^ revealed that the introduction of PEG segment was beneficial to the formation of SC crystals and improvement of crystallization rate in PLLA/PDLA–PEG–PDLA.

Despite those recent studies, it is still unclear how the SC crystals and flexible PEG blocks affect the structure and properties of PLLA, especially the structural evolution of those materials under deformation. Herein, PLLA/PLLA–PEG–PLLA, PLLA/PDLA, and PLLA/PDLA–PEG–PDLA blends were selected for a systematic study on the effect of PDLA and PEG on the crystalline structure and kinetics, tensile properties and deformation mechanism of those materials. Different stretching temperatures were selected, covering the range of room temperature to 80 °C, considering the application environments as plastics.

## Experimental section

### Materials

PLLA (optical purity = 99.7%, *M*_n_ = 95 kg mol^−1^, PDI = 1.43) was obtained from Zhejiang Haizheng Biomaterials Co., Ltd. PDLA (*M*_n_ = 12 kg mol^−1^ and PDI = 1.16) and PLLA_5k_-*b*-PEG_4k_-*b*-PLLA_5k_ and PDLA_5k_-*b*-PEG_4k_-*b*-PDLA_5k_ were purchased from Jinan Daigang Biomaterial Co., Ltd. Antioxidant (1010) was purchased from BASF Co., Ltd.

### Samples preparation

All the polymer samples were dried at 80 °C in a vacuum oven for 24 h. The composites were prepared by melt extrusion with an internal mixer (Haake Rheomix OS) at 190 °C with a rotating speed of 50 rpm for 5 min. The weight ratio of PLLA/PDLA, PLLA/PLLA–PEG–PLLA, and PLLA/PDLA–PEG–PDLA blends was fixed to 90/10 (Table 1). 0.5 mm thick plates of neat PLLA and its blends were prepared using a compression molding machine at a temperature of 190 °C (for neat PLLA) or 230 °C (for PDLA-containing blends). The molding pressure was 40 MPa and the pressure holding time was 3 min. Subsequently, the samples were cooled down quickly to room temperature in an ice-water bath. The specimens were cut into dumbbell shape with 40 mm in length, 2 mm in width, and 0.5 mm in thickness.

**Table 1:** Composition information of PLLA and its blends

Samples	PLLA/wt%	PDLA/wt%	PEG/wt%
PLLA	100	—	—
PLLA/PLLA–PEG–PLLA	90	—	2.86
PLLA/PDLA	90	7.14	—
PLLA/PDLA–PEG–PDLA	90	7.14	2.86

### Measurement and characterization

The melting and crystallization behavior of PLLA and the blends were performed with a TA Q2000 DSC (TA Instruments, USA) under nitrogen atmosphere at 50 mL min^−1^. The instrument was calibrated with indium before measurements. About 5–10 mg samples were weighed and sealed in an aluminium pan, heated from 30 °C to 250 °C and held for 3 min to eliminate the thermal history. The samples were first cooled to 30 °C at 10 °C min^−1^, and then reheated to 250 °C at 10 °C min^−1^.

The spherulitic morphologies of PLLA and the blends were observed with a polarized optical microscope (Olympus BX51, Japan) equipped with a Linkam hot stage. The samples were sandwiched between two glass plates, kept in the melt at 230 °C for 3 min to eliminate the thermal history. Then, the specimens were cooled to 120 °C at a cooling rate of 60 °C min^−1^ for isothermal crystallization.

X-ray diffraction (XRD) measurements were carried out using a Bruker AXS D8 diffractometer with Cu Kα radiation (*λ* = 1.54 Å). The 2D-WAXD patterns were collected by the two-dimensional detector VANTEC-500. The exposure time was 300 s and the sample-to-detector distance was 85.6 mm.

The samples were stretched on a Linkam TST 350 hotstage (Linkam Scientific Instruments, Ltd., U.K.) at 30 °C, 40 °C, 45 °C, 55 °C, 58 °C and 80 °C, respectively. The crosshead speed was 150 μm s^−1^. Engineering stress (*σ*) and engineering strain (*ε*) were calculated according to *σ* = *F*/*A* and *ε* = (*L* − *L*_0_)/*L*_0_ respectively, where *F* is the force, *L* is the sample length, *L*_0_ is the initial sample length and *A* is the initial cross section area.

*In situ* X-ray scattering measurements were carried out at 80 °C. The patterns were collected at every 2 mm until the mini tensile bars were stretched to 200% strain. The image acquisition time was 40 s. All the images were corrected for background scattering, air scattering, and absorption. Two-dimensional patterns were converted into one-dimensional scattering curves along the angle 0–360°. The relative fraction of different phases was calculated according to:1
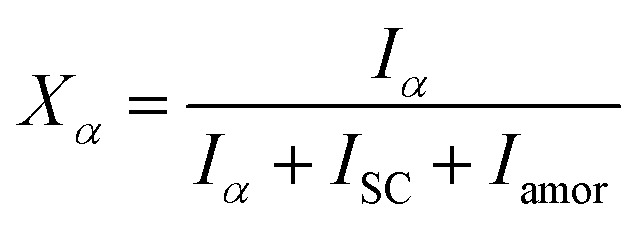
2
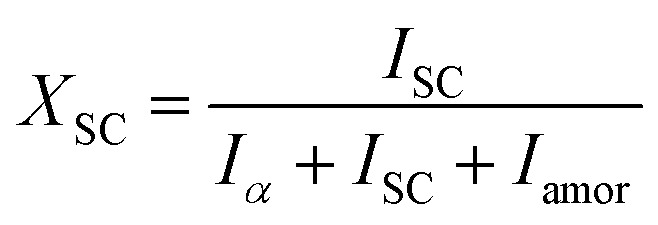

3 *X* = *X*_α_ + *X*_SC_

where *X*_α_, *X*_SC_, and *X* represent the phase content of α crystals, SC crystals and overall crystallinity, respectively. *I*_α_ and *I*_sc_ stand for the sum of the integrated scattering intensity of the reflections of α crystals and SC crystals. *I*_amor_ indicates the integrated intensity of the amorphous halo. The quantitative degrees of orientation of the lattice plane (200)/(110) in uniaxially oriented samples were calculated according to the Hermans formula:^29,30^
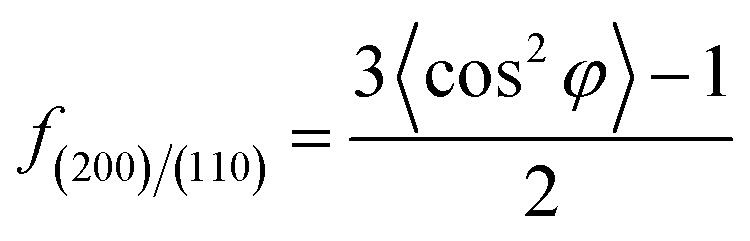
where, *φ* is the angle between the normal direction of the crystal plane and the reference axis. 〈cos^2^ *φ*〉 is defined as:
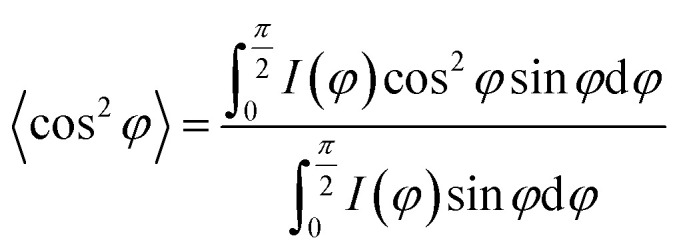
where, *I*(*φ*) is the scattering intensity along the angle *φ*.

## Results and discussion

### Crystallization behavior

**Fig. 1:**
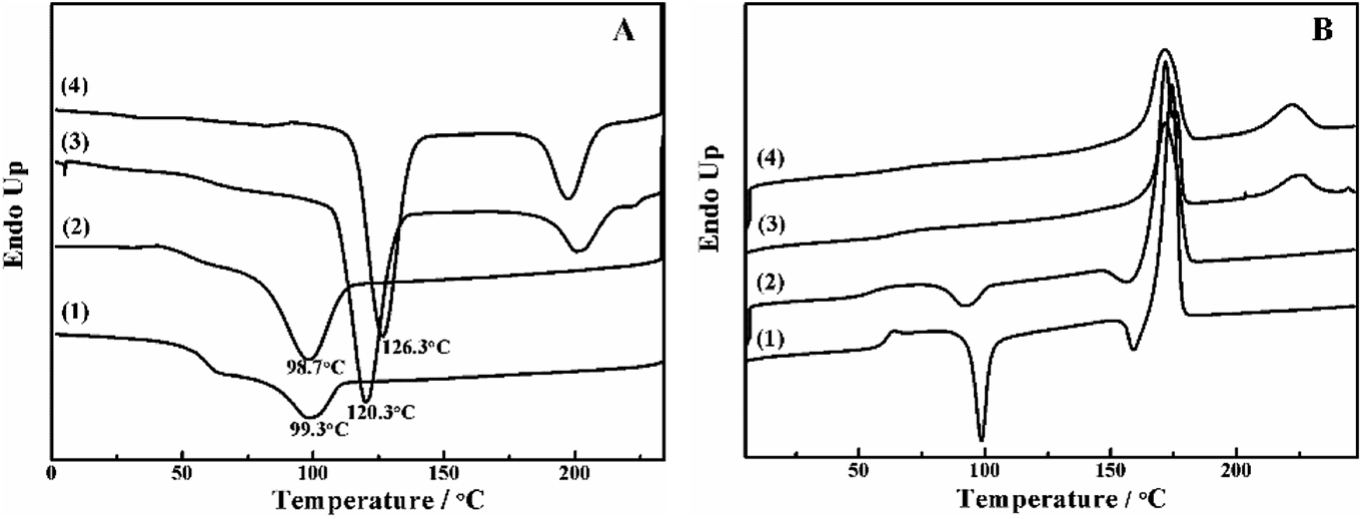
DSC curves of PLLA (1), PLLA/PLLA–PEG–PLLA (2), PLLA/PDLA (3) and PLLA/PDLA–PEG–PDLA (4) blends in (A) the cooling and (B) the second heating processes. The heating and cooling rate was 10 °C min^−1^.

The crystallization behavior of PLLA and its blends are investigated by DSC. As shown in Fig. 1A, the crystallization temperature of PLLA/PDLA appears at 120.3 °C, which is higher than that of neat PLLA and PLLA/PLLA–PEG–PLLA blend. Besides, a new peak appears at ∼220 °C, which indicates the formation of SC crystals in PLLA/PDLA. The presence of SC crystals acts as heterogeneous nucleating seeds for the homocrystals of PLLA. Furthermore, the crystallization temperature of the homocrystals in PLLA/PDLA–PEG–PDLA blend increases further to 126.3 °C. The subsequent heating curves of PLLA and PLLA/PLLA–PEG–PLLA show a baseline shift and an endothermic peak located at 58 °C and 175 °C, corresponding to glass transition and melting of α/α′ crystals. Besides, two exothermic peaks at 95 °C and 157 °C can be assigned to cold crystallization and the transition from α′ to α phase^31,32^ respectively (Fig. 1B). For PLLA/PDLA and PLLA/PDLA–PEG–PDLA blends, the cold crystallization peak and the α′ to α transition peak disappear, while the endothermic peak of SC crystals appears, indicating that crystallization is more complete in the PDLA-containing samples during cooling and the more stable α crystals are formed.

**Fig. 2:**
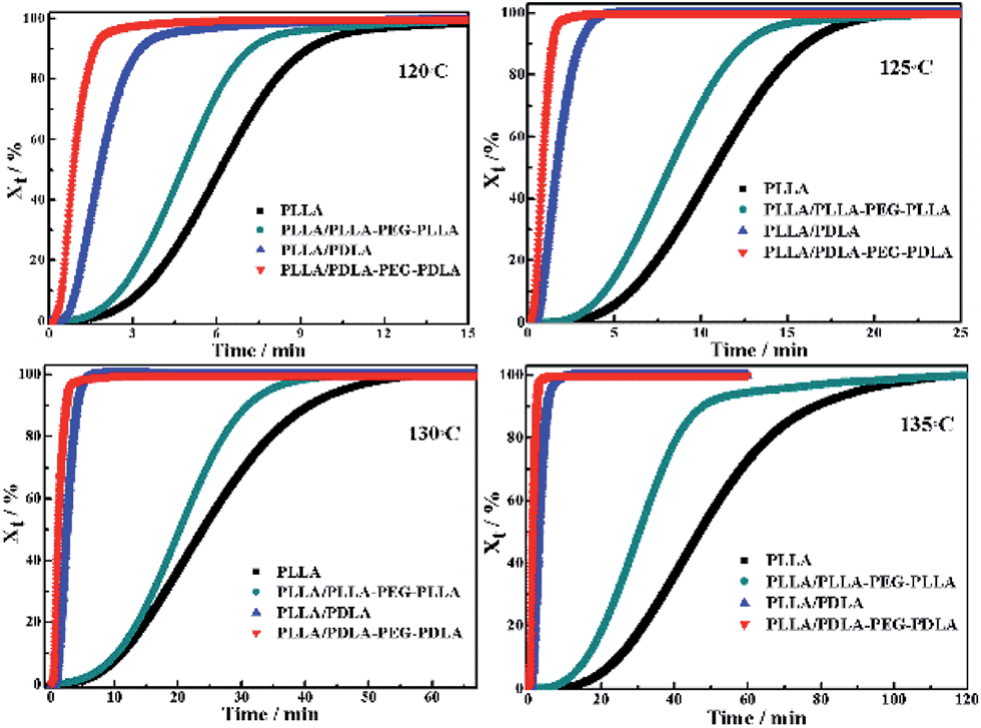
The relative crystallinity (*X*_t_) of PLLA and blends isothermally crystallized at different temperatures as a function of crystallization time.

**Fig. 3:**
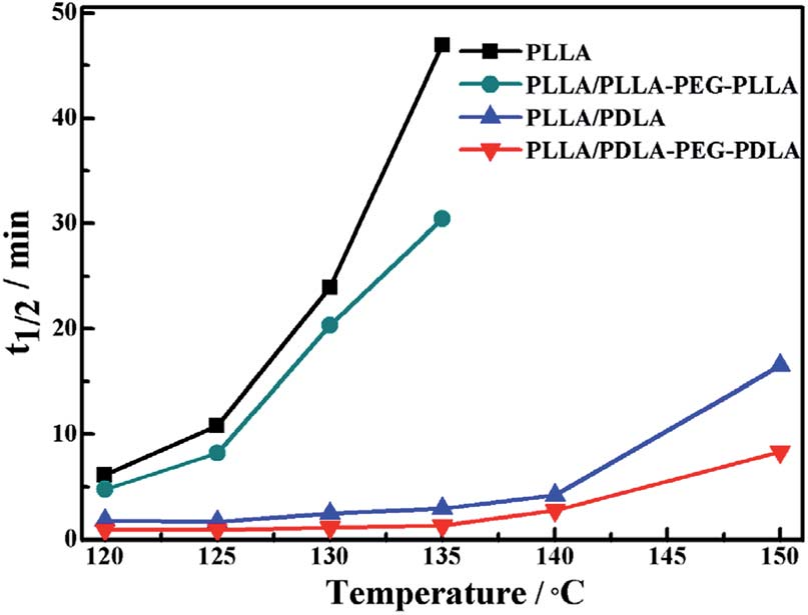
The half-crystallization time of PLLA and blends isothermally crystallized at different temperatures.

The isothermal crystallization kinetics of samples at the temperature ranges of 120–150 °C are further studied by DSC (Fig. 2). For all the four selected temperatures, the crystallization time decreases with the sequence of PLLA > PLLA/PLLA–PEG–PLLA > PLLA/PDLA > PLLA/PDLA–PEG–PDLA. Furthermore, the crystallization time of the samples gradually increases with crystallization temperature. Moreover, the half-crystallization time of pure PLLA and PLLA/PLLA–PEG–PLLA increases more sharply than that of PLLA/PDLA and PLLA/PDLA–PEG–PDLA blends (Fig. 3). When the crystallization temperature is above 140 °C, it is difficult to nucleate in the PLLA and PLLA/PLLA–PEG–PLLA blends and the crystallization rate is too slow to be measured. The PEG in the PLLA–PEG–PLLA and PDLA–PEG–PDLA can act as a plasticizer to accelerate the crystallization of PLLA homocrystals. Besides, the formation of SC crystals after blending PDLA is beneficial to the crystallization of PLLA homocrystals by heterogeneous nucleation effect.

**Fig. 4:**
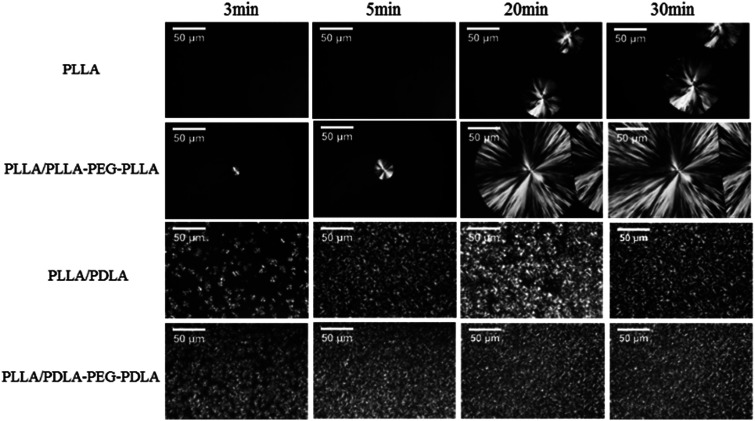
POM images for PLLA and its blends isothermally crystallized at 120 °C for different time.

**Fig. 5:**
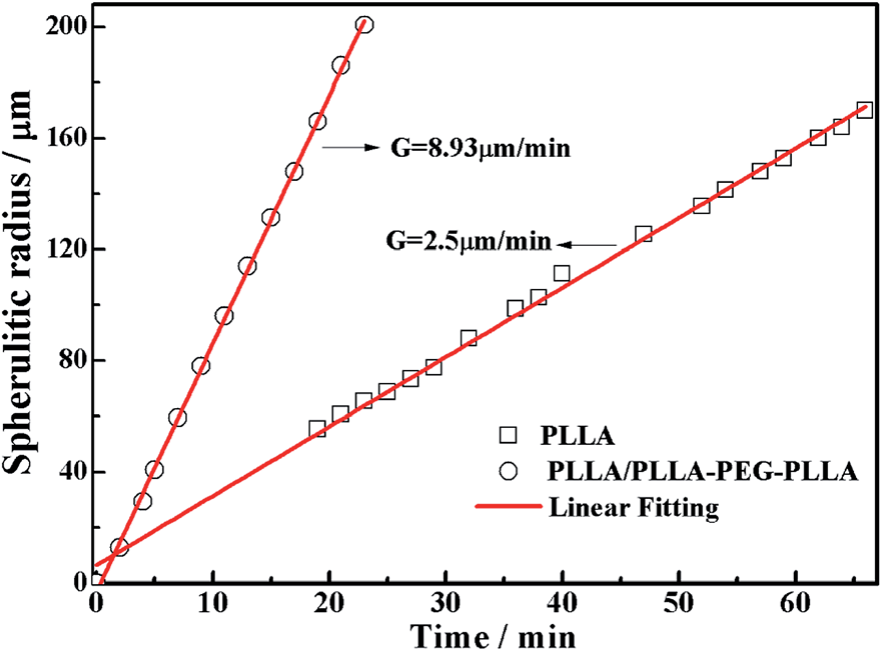
Plots of spherulitic radius against time during isothermal crystallization at 120 °C.

All specimens after isothermal crystallization at 120 °C exhibit typical spherulitic morphology (Fig. 4). The nucleation densities of PLLA and PLLA/PLLA–PEG–PLLA are relatively small. As shown in Fig. 5, the spherulitic growth rate is higher when PEG segments are introduced into the system, possibly because the chain mobility of PLLA is increased.^33^ Tiny crystals are observed in PLLA/PDLA and PLLA/PDLA–PEG–PDLA blends, indicating larger nucleation densities. Besides, the spherulitic density of the PLLA/PDLA–PEG–PDLA is higher than that of PLLA/PDLA.

### Tensile properties of the compression molded specimen

**Fig. 6:**
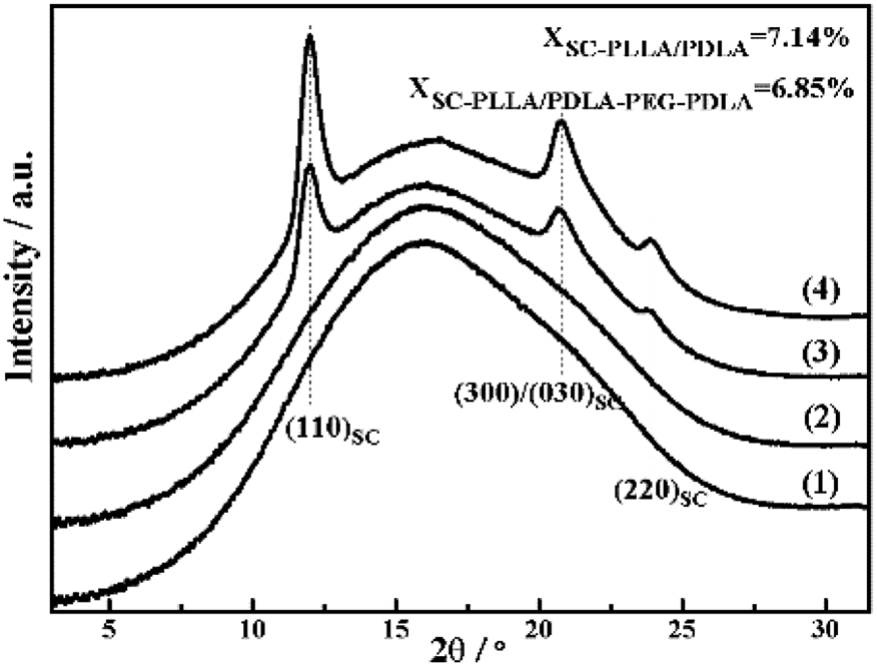
1D-WAXD intensity profiles of initial PLLA (1) and PLLA/PLLA–PEG–PLLA (2), PLLA/PDLA (3) and PLLA/PDLA–PEG–PDLA (4) blends.

The WAXD intensity profiles of the compression molded specimen are displayed in Fig. 6. The pure PLLA and PLLA/PLLA–PEG–PLLA are amorphous and no diffraction peak is visible. In contrast, the PLLA/PDLA and PLLA/PDLA–PEG–PDLA blends exhibit three peaks located at 2*θ* = 11.9°, 21°, and 23°, respectively, which correspond to the (110), (300)/(030) and (220) of SC crystals.^34^

**Fig. 7:**
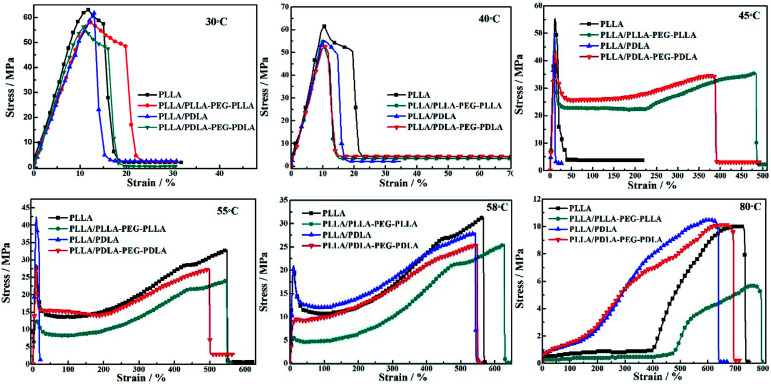
Engineering stress–strain curves of PLLA and blends stretched at different temperatures. The stretching rate was 150 μm s^−1^.

Stress–strain curves of the polymer stretched at different temperatures are shown in Fig. 7. The polymer chains of PLLA are virtually frozen when the stretching temperature is well-below the *T*_g_ of PLLA (30 °C and 40 °C), resulting in poor tensile properties and brittle fracture. When the stretching temperature increases to 45 °C, the necking phenomenon appears and the elongation at break of PLLA/PLLA–PEG–PLLA and PLLA/PDLA–PEG–PDLA blends increases up to 480% and 390%, respectively. It can be attributed to the plasticization effect of PEG segments in those samples, *i.e.*, the PEG flexible chains can act as a plasticizer to facilitate the movement of PLLA chains. When the stretching temperature is increased to 55 °C, the polymers, except for PLLA/PDLA blend, show very high elongation. However, PLLA/PDLA has the highest yield strength as compared with other samples, which can be ascribed to the presence of SC crystals in PLLA/PDLA. These results suggest that both SC crystals and soft segments affect the tensile properties of PLLA. When the temperature is close to *T*_g_ of PLLA (58 °C), all the samples exhibit typical necking phenomenon. When the temperature is further increased to 80 °C, the PLLA chains are in the rubbery state, therefore, no yielding is observed and strain-induced crystallization occurs. The introduction of SC crystals and soft segments affects the microstructure formation mechanism and in turn, influences the mechanical properties.

### *In situ* WAXD during stretching

**Fig. 8:**
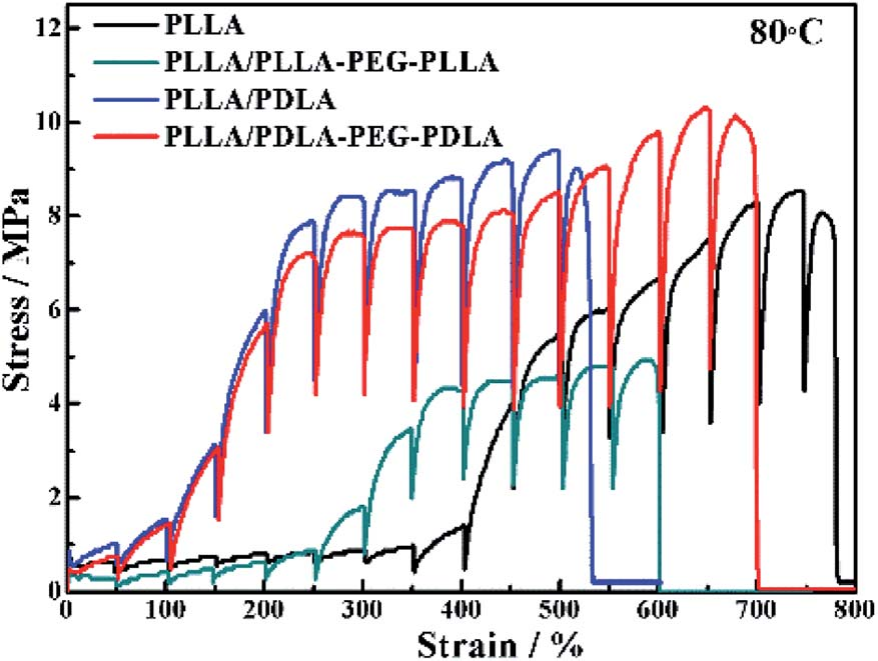
Engineering stress–strain curves of PLLA and blends stretched at 80 °C. The stretching rate was 150 μm s^−1^.

The *in situ* WAXD technique is applied to further explore the microstructure evolution of the samples during stretching at 80 °C. The stress–strain curves of pure PLLA and its blends during the *in situ* WAXD measurement are shown in Fig. 8. The pure PLLA displays a rubber-like behaviour at low strain (<400%) and a low elastic modulus. The stress of PLLA increases slightly with the increase of strain. The stress of PLLA/PLLA–PEG–PLLA and PLLA/PDLA blend increases sharply when the strain reaches 300% and 100%, respectively. The introduction of soft segments can improve the elongation at break from 540% (PLLA/PDLA) to 700% (PLLA/PDLA–PEG–PDLA). There is some slight difference between Fig. 7 and 8 due to the annealing effect. However, the general features are the same.

**Fig. 9:**
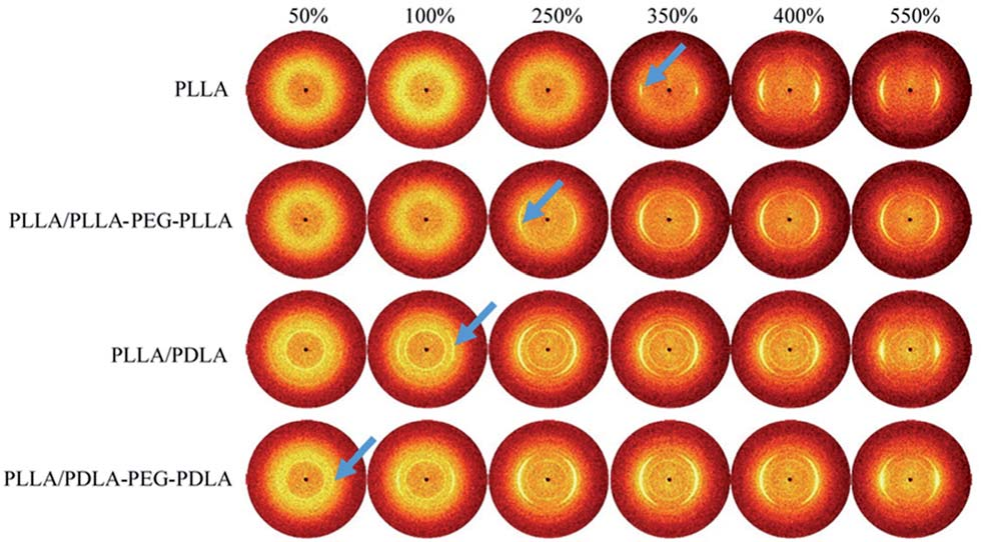
WAXD patterns of PLLA and the blends with different strains at 80 °C. The stretching direction was vertical with a speed of 150 μm s^−1^. Arrows indicate the diffraction of homocrystals.

**Fig. 10:**
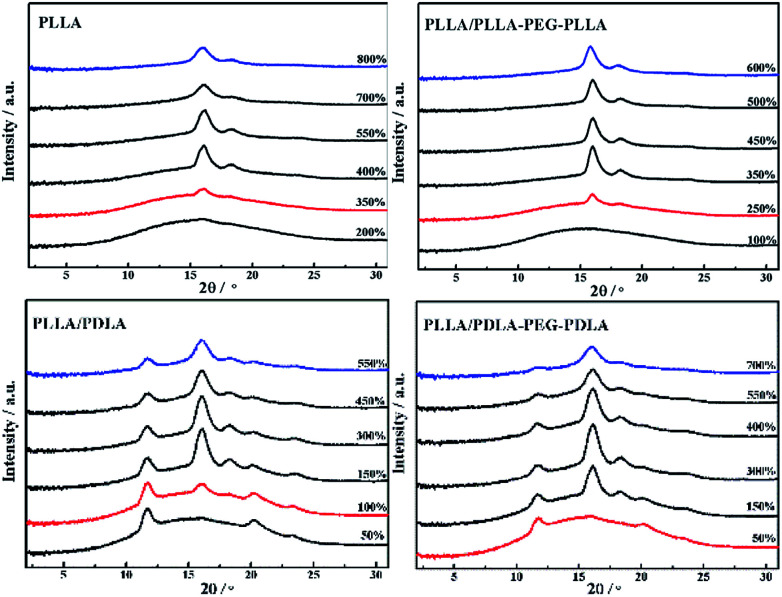
1D-WAXD intensity profiles of PLLA and blends stretched at 80 °C. The stretching speed was 150 μm s^−1^.

2D-WAXD patterns and the corresponding 1D WAXD intensity profiles for different strains during stretching are shown in Fig. 9 and 10, respectively. New diffraction arcs appear in the equatorial direction with the increasing strain, which is assigned to the (200)/(110) and (203) crystal planes of PLLA homocrystals. The critical strains for homocrystal formation are 350% for pure PLLA, 250% for PLLA/PLLA–PEG–PLLA, 100% for PLLA/PDLA, and 20% for PLLA/PDLA–PEG–PDLA, respectively. These results indicate that the PEG and SC crystals can promote the crystallization of PLLA both under static condition and during stretching.

**Fig. 11:**
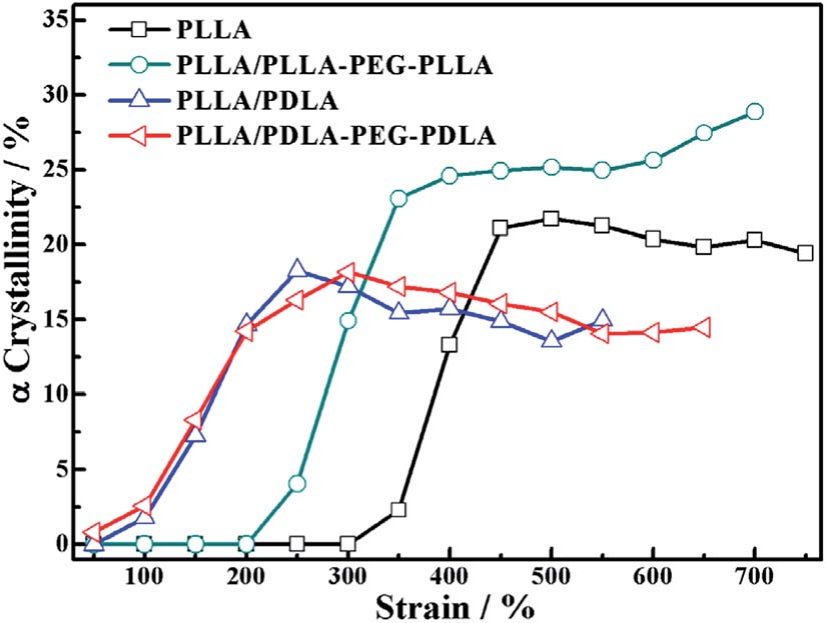
The variation of α crystallinity of specimens as a function of strain during stretching at 80 °C.

Because of the different critical strains of α crystals during stretching, the structure under the same strain condition is different, which results in various stress (Fig. 11). The contents of α crystals increase rapidly at low strains due to the presence of SC crystals in PLLA/PDLA and PLLA/PDLA–PEG–PDLA blends. The final content of α crystals of PDLA-containing blends is lower than those of pure PLLA and PLLA/PLLA–PEG–PLLA. It can be ascribed to the fact that a fraction of the PLLA chains is consumed by SC crystals.

**Fig. 12:**
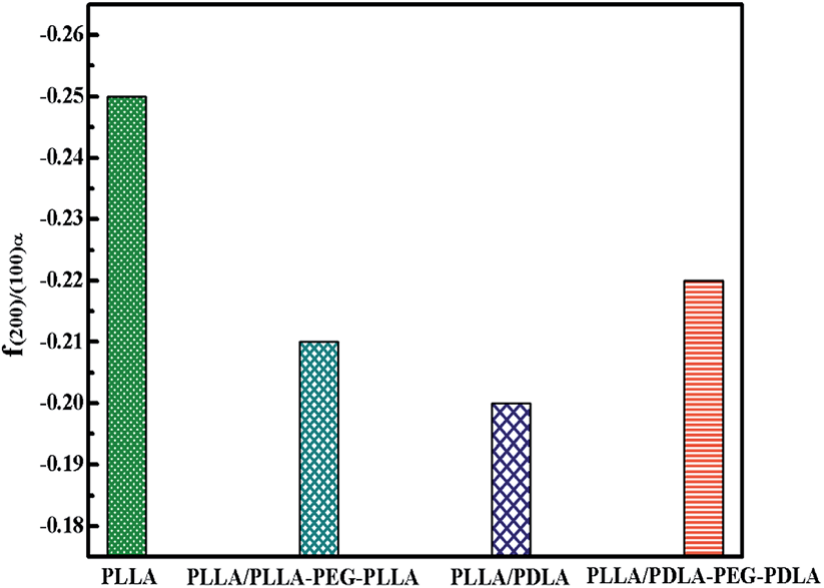
The orientation parameter of (200/110)_α_ crystal plane for neat PLLA and its blends after uniaxial stretching at 80 °C, respectively.

The orientation parameters of (200)/(110)_α_ crystal plane of stretched samples are shown in Fig. 12. It is easier to reach higher orientation when the crystallization of PLLA occurs at higher strains. Therefore, the pure PLLA has the highest degree of orientation. The introduction of PLLA–PEG–PLLA, PDLA and PDLA–PEG–PDLA reduce the critical strain of crystallization, resulting in a lower orientation of α crystals. In addition, the low fracture strains of PLLA/PDLA, and PLLA/PLLA–PEG–PLLA blends also lead to lower final crystal orientation.

### Crystallization after step-strain

**Fig. 13:**
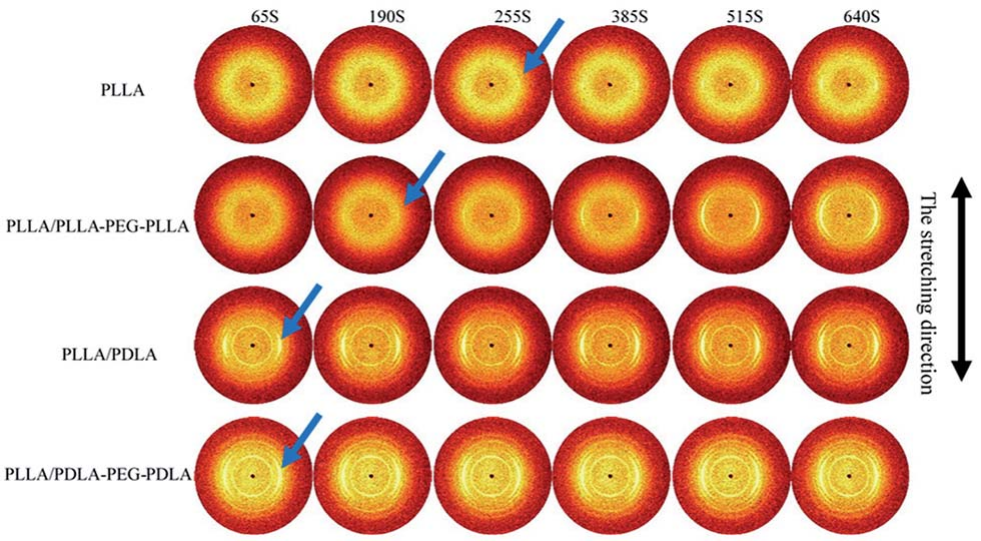
Selected 2D WAXD patterns of pure PLLA and blends during isothermal crystallization at 80 °C after uniaxial stretching to 200%. The stretching speed was 150 μm s^−1^. The stretching direction was vertical.

**Fig. 14:**
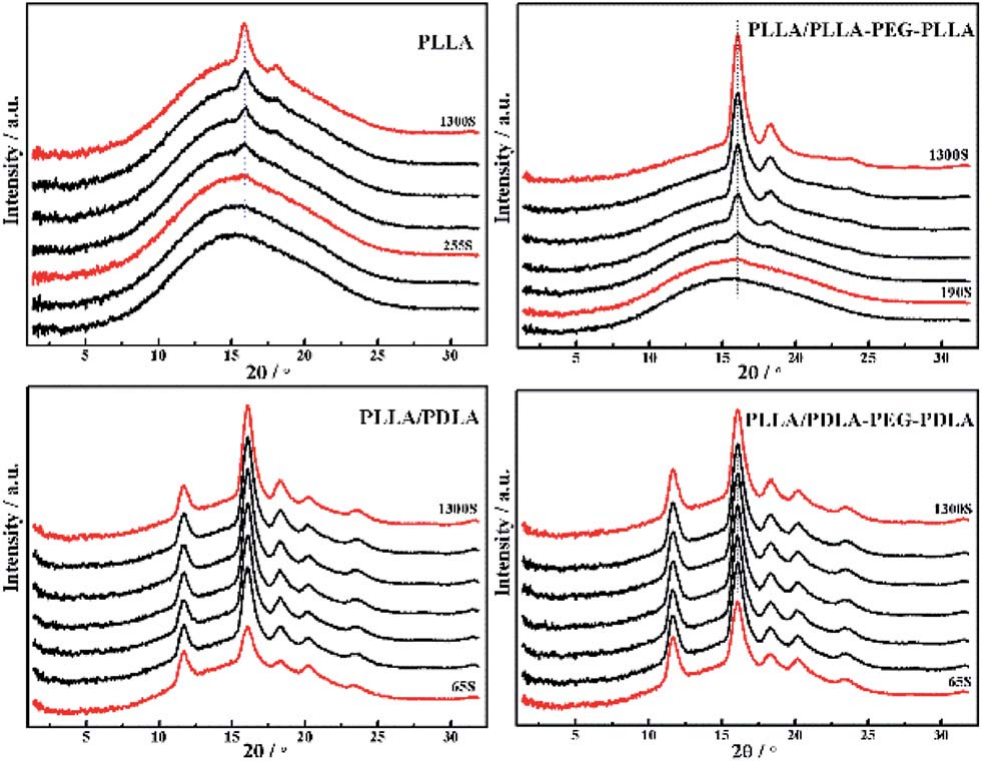
1D WAXD intensity profiles of PLLA and blends during isothermal crystallization at 80 °C after uniaxial stretching to 200%.

To further investigate the effect of SC crystals and flexible segments on the crystallization of specimens, *in situ* WAXD with step-strain deformation are measured (Fig. 13). The samples are stretched to a constant strain of 200% at a stretching rate of 150 μm s^−1^. The patterns in the first column show that neat PLLA and PLLA/PLLA–PEG–PLLA are amorphous after stretched to 200%. After 190 s, a weak (110/200)_α_ diffraction arc appears in PLLA/PLLA–PEG–PLLA, slightly earlier than the onset time for PLLA (255 s), indicating that the PEG facilitates PLLA crystallization. The PLLA/PDLA and PLLA/PDLA–PEG–PDLA show strong (200/110)_α_ and (203)_α_ reflections immediately after stretching. More detailed information is shown in the 1D WAXD intensity profiles (Fig. 14).

**Fig. 15:**
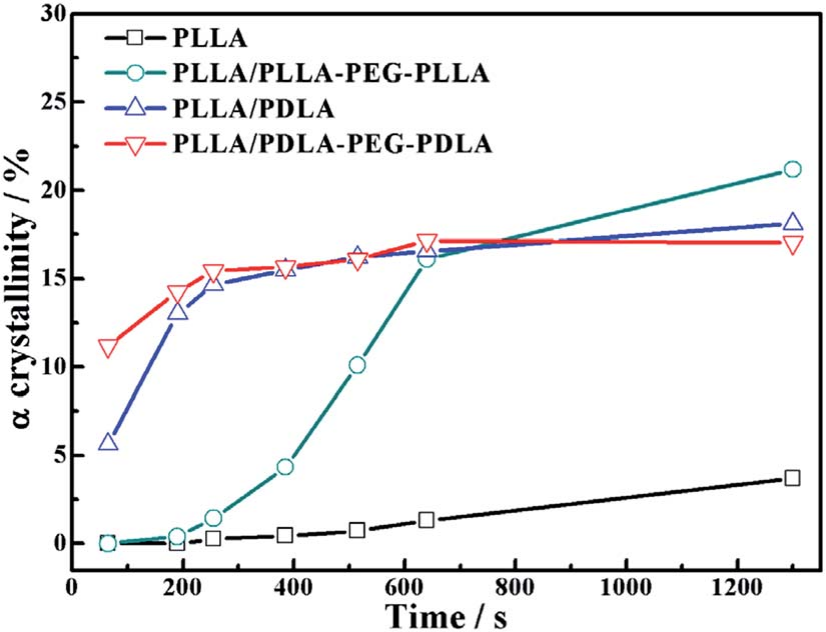
The variation of α crystal in neat and blends as a function of time during isothermal process at 80 °C after step-stretching to 200%. The stretching speed was 150 μm s^−1^.

**Fig. 16:**
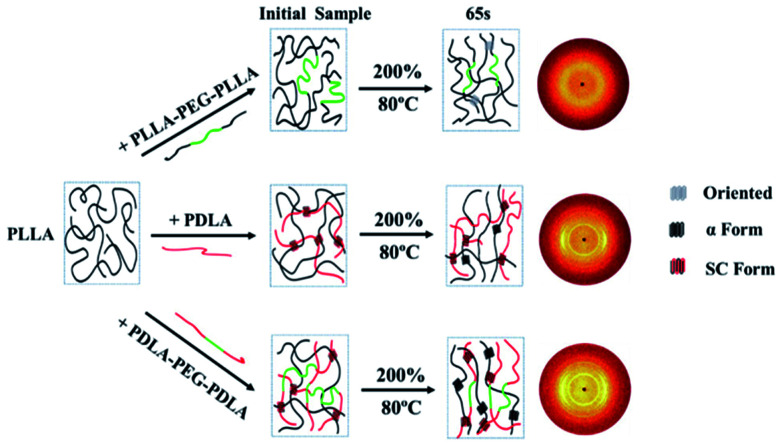
Schematic illustration of structural evolution of PLLA and blends under 80 °C after step-strain.

As shown in Fig. 15, the crystallinity of PLLA/PDLA–PEG–PDLA is 11.18% at 65 s, which is higher than that of PLLA/PDLA (5.62%). All the above results indicate that both the SC crystals and PEG blocks contribute to the crystallization of PLLA. Based on the above results, a schematic illustration of the effect of SC crystals and flexible PEG blocks on PLLA crystallization under strain is depicted in Fig. 16. The PLLA and the PLLA/PLLA–PEG–PLLA blend are amorphous, while SC crystals are present in the PDLA containing blends (PLLA/PDLA and PLLA/PDLA–PEG–PDLA). When stretched to 200% at 80 °C, no α crystals are detected in the PLLA/PLLA–PEG–PLLA blend in the beginning (after 65 s). However, due to the nucleation effects of SC crystals, the PLLA/PDLA blend and the PLLA/PDLA–PEG–PDLA blend form α crystals immediately, with the same thermal and deformation history. The crystallization rate is the highest for the PLLA/PDLA–PEG–PDLA blend because of the coexistence of nucleation agent and plasticizer. With the same processing condition, a higher crystallization rate will generally result in a higher crystallinity, which will impart the materials with higher stiffness and strength. The flexible PEG blocks could act as “molecular lubricants”, thus increasing the toughness of the PLLA blend.

## Conclusion

In this work, by adding ABA block copolymers, the effect of flexible PEG blocks and PDLA chains on the crystallization and mechanical properties of PLLA was studied by DSC, POM, and WAXD. It was observed that SC crystals promote the nucleation of α crystals and PEG blocks attribute to a higher spherulitic growth rate. With the combination of PEG and PDLA chains, the crystallization temperature of PLLA/PDLA–PEG–PDLA increased significantly. The elongation at break of PLLA/PDLA–PEG–PDLA could reach 400% with the help of PEG segments at temperatures higher than 45 °C, while the stiffness was largely maintained due to the enforcing effect of SC crystals. By *in situ* WAXD during stretching, the critical strains of α crystal formation at 80 °C in different samples decreased in the following order: PLLA > PLLA/PLLA–PEG–PLLA > PLLA/PDLA > PLLA/PDLA–PEG–PDLA. During isothermal crystallization after step-strain, the content of α crystals of PLLA/PDLA–PEG–PDLA was significantly higher than that of other blends at 65 s. All the results suggested that the coexistence of SC crystals and soft chains could effectively improve the toughness and crystallization rate of PLLA, thus enhancing the comprehensive properties of PLLA.

## Conflicts of interest

There are no conflicts to declare.

## Supplementary Material
